# Criminal Abortion

**Published:** 1857-08

**Authors:** 


### *Criminal Abortion.*

We have watched with much interest the progress of the movement lately commenced by the profession in Boston against Criminal Abortion. This movement has now secured the sanction of the State Medical Society of Massachusetts, and as it will doubtless be participated in by physicians of other States, we are inclined to think its history from the outset, obtained from current numbers of the Boston Medical and Surgical Journal, and from the correspondence of a friend may prove not unacceptable to our readers.

That this crime, terrible and infamous though it be, is yet common, none who have watched the proceedings of the Courts, or have read the daily journals can doubt—and there is every reason to believe that the community on the one hand are too often ignorant of its true character, and on the other too prone to the opinion that it is frequently practised, advised or excused by respectable medical men. To meet these errors and to vindicate the profession has now been attempted. More than two years and a half ago, in November 1855, the Professor of Obstetrics in Harvard University, (Dr. Storer, sen.), in the Public Address introductory to the Annual Lectures, took occasion to call attention to the prevalence in this country of Criminal Abortion, and the equally injurious and immoral practice of preventing pregnancy.

The sentiments then expressed were received with approbation, and were requested for publication. Fearful however lest its pecuniary interest might suffer, whether at the hands of the public, one of whose prevailing sins had been so boldly and directly assailed, or of the profession, whose lethargy in this matter and subservience to expediency had been rebuked, the college interfered, and at the express desire of its faculty, but, as we now learn, entirely against the author's will, such portions of the address as bore on Criminal Abortion were suppressed in the published copy.

With the exception of a similar address by Dr. Hodge of Pennsylvania, no other public action against the crime of abortion, seems to have been taken until now, by any of the profession in this country.

In February of the present year, Dr. Storer, Jr., (Horatio R)., of Boston, having ascertained that his father had abandoned all intention of prosecuting his original proposal, brought the subject before the Suffolk District Medical Society. In a few preparatory remarks:

"Dr. Storer quoted statistics from a recent memoir by Tardieu of Paris in the Annales d'Hygiene Publique et de Med. Legale for 1866, showing how common was the crime in this country as compared with others; so common indeed as to have led foreigners to suppose that the procuring criminal abortion was with us an ordinary and well established branch of industry, not interfered with by the Law; as indeed to all intents and purposes is at present the case.

Dr. S. referred to our statutes on this subject and to the ignorance prevalent in the community respecting the actual and separate existence of fœtal life in the early months of pregnancy. He dwelt on the moral and absolute guilt of the parties offending and on the necessity of prompt and efficient action by the profession, and called upon the Society, as representing the physicians of Boston, to take such steps as would alike ensure the innocence in this matter of all its members, and show to the community the sincere abhorrence with which they viewed the crime.

In conclusion Dr. Storer offered the following resolution:—'That a committee be appointed to consider whether any further legislation is necessary in this Commonwealth on the subject of Criminal Abortion, and to report to the society such other means as may seem necessary for the suppression of this abominable, unnatural and yet common crime.'

To which, at the suggestion of Dr. Jeffries, was added this amendment:—'And that said report when accepted by this Society shall by it be recommended to the Massachusetts Medical Society, as a basis for its further action.'"

The resolution and amendment were unanimously passed, and Dr.'s H. R. Storer, Bowditch and Ellis appointed as the Committee.

At the April meeting the committee submitted their report, which was ordered to be printed. It gave rise to much discussion, both in private and at a special meeting of the Society called early in May; part in the debate being taken by most of the leading practitioners of the City.

The Committee, after due deliberation and under advice of eminent

Criminal Counsel, were of opinion that under the existing law of Massachusetts convictions were impossible, and they therefore reported a draft in substitute for the present statutes, during the preparation of which "they had addressed letters of inquiry to distinguished Physicians in every part of the Union, and received answers relative to the statutory laws of twenty-one States, accompanied in several instances by the opinions of leading advocates and Judges, for them procured."

The report itself was acknowledged to be able and forcible, but to the draft many objections were made. Gentlemen of the timid or ultra-conservative schools were not prepared to meet the issue face to face; and to ensure as much unanimity as possible in the matter, the Committee, by permission of the Society withdrew this portion of the report, which as thus amended by them was accepted at the regular May meeting, and the following resolution passed:—

*Resolved*, That the subject of Criminal Abortion demands the attention of the Medical Profession of the State.

*Resolved*, That the Massachusetts Medical Society be urged to take action in the premises, and, if it deem expedient, to present the subject for the consideration of the Legislature.

In persuance of this latter resolve, the same Committee by direction of the Society, brought the matter on June 3rd, before the Massachusetts Medical Society at its annual meeting at New Bedford.

By special vote of the Society, its rules were suspended, and:—

"The debate was warmly and ably sustained for about an hour and a half, the principal speakers being Dr.'s H. R. Storer, J. Bigelow, J. Ware, and Ephraim Buck, of Boston, C. C. Chaffee, of Springfield, and Henry H. Childs of Pittsfield. The resolutions offered by the Committee were finally referred to a Committee consisting of Messrs. F. Hooper of Fall River; J. Bigelow, J. Ware, Chas. Gordon and H. R. Storer, of Boston; J. C. Dalton of Lowell and E. Hunt of Danvers, who are to report to the Councillors at their next meeting."

The discussion of this question has thus fairly begun, and the attention of the Profession will undoubtedly be engrossed by it throughout the land. As we are in possession of a copy of the Committee's report, we shall freely quote their argument:—

"The frequency of Criminal Abortion in Massachusetts though already notorious, is much greater than is generally supposed, and it is probably steadily increasing. It is confined to no class of persons, neither the poor, the ignorant nor the unmarried; indeed among the married, the crime seems more common than among those who have the excuse of shame.

These statements might be doubted by gentlemen, who, for whatever reason, do not happen to be personally familiar with the fact. They are nevertheless strictly true. Statistics of abortion do not exist, and for evident reasons, reliable ones cannot be obtained. The instances of its occurrence, even when accidental, in advanced pregnancy, are but seldom reported to the proper authorities, who acknowledge that many of the so-called still-births, are in consequence of induced abortion. 'Undoutedly more than a hundred such, yearly escape being recorded (in Boston), a large proportion of which no doubt result from criminal abortion.' (Extract from a letter of the City Registrar, Mr. Apollonio, to the Committee, March 26, 1857.)

Common as the crime may be in an advanced stage, after the existence of pregnancy has been made certain by its more evident signs, it is infinitely more common in the early months, before motion has occurred, when so many believe that the child has not yet received life.

It is impossible for the Committee to furnish statistics in this matter; but lest their premises should be denied, and as the experience of a single individual, it may be remarked that in no less than *fifteen* instances during the past half-year has the chairman been called to treat the *confessed* results, near or remote, of criminal abortion; and of these patients, all without exception were married and respectable women. This very prevalence of the crime, which has hitherto obtained to an equal extent only in barbarous or heathen nations, is found almost to protect it.—It escapes punishment by law. Many cases in proof of this might be instanced from the records of our courts; the three given in the City Registrar's report for the past year, (page 18), will, however, suffice. It has found public and unblushing defenders, who have so blunted the moral and religious sense of the people, that many respectable women do not hesitate to avow their belief that abortion is no crime.

The impunity with which the crime is committed is mainly owing to four different and separate causes:—

1st. The present *morale* of the community in reference to the subject;

2nd. The great caution generally observed by the perpetrators of the crime;

3rd. The fact that both operator, where such has been employed, and patient, are extremely desirous of concealment, and so can seldom be produced as witnesses; women frequently inducing criminal abortion upon themselves, either by drugs, over-exertion or instruments, without the knowledge, advice, or aid of others; and

Finally, The defective character of the law itself.

To check this evil, a change must be effected in public opinion. By whom shall this change be begun? There is no use in longer silence or in attempting to conceal what is but too evident. It has clearly become the duty of the Medical profession, as the guardians of the public health, if for no higher reason, and as those who, of all others, sooner or later in almost every instance become cognizant of the crime, to declare its true nature, its prevalence, and deplorable consequences; to denounce it in unmeasured terms, and, where possible, to point out and to enforce efficient means for its suppression.

The ways in which the popular ignorance respecting its actual guilt, its immediate dangers to the mother, and its effects on subsequent health, can best be enlightened, are self-evident. In private, among his families; in public from his professor's desk, from the pages of his journal, or from the witness' stand, the physician is called upon by every dictate of humanity aud religion, to comdemn it.

That the Massachusetts law is predicated on an entirely erroneous idea of the true nature and guilt of criminal abortion, is evident from its very face. The crime is here considered as one against the person of the women, and against her alone. If she die in consequence of the abortion, a certain penalty is provided; if she do not die in consequence thereof, though the abortion be fully accomplished, a comparatively trifling punishment is inflicted, less even than that ordinarily given to simple maim or mutilation.

The error is one of intent, which in reality is seldom against the life of the mother; in almost every instance, she is herself, not merely an attempt at the murder of herself, for that would be simply absurd, but the murder of her child.

The law is here fundamentally wrong. It utterly ignores the existence of the living child, though the child is really alive from the very moment of its conception, and from that very moment is and should be considered a distinct being; this the law does not, however, recognize. The fœtus is not as has been so often alleged, merely *pars viscerum matris*; though upon such belief our law is evidently based.

'It would be hard,' writes an eminent lawyer of this city to the Committee, 'to find in the moral law the distinction made in the civil law, between causing the death of a child before and immediately after his birth. Before the birth, though the civil law recognizes the existence of the child for some purpose, still so far as personal injury is concerned, its being is engrossed, so to speak, in the mother.'

The argument that the perils and dangers to which the fœtus is naturally subjected during its intra-uterine life should lessen the criminality of attempts at its destruction, is unworthy the profession, and should not be allowed in law. These perils do not exceed, if indeed they equal, those which threaten the new-born child. Yet these last would hardly be allowed to invalidate a charge of infanticide.

The crime, when a living fœtus has been destroyed, is clearly murder. But as there are those who will not allow that it should be punished as such, it should in all cases, whether the mother lives or not, be regarded as at least felony by the law, which now in most cases, declares it but a simple misdemeanor. In cases where both lives are lost, its guilt is proportionally increased. If wilful intent against the life of the mother thus destroyed could ever be proved, the indictment should then be for murder.

An article of the penal code of France, as drawn by Napoleon, provides an increase of punishment where criminal abortion shall have been induced or advised by physicians, surgeons or other officers of health, or by druggists. We may well imitate this example. If it be determined that the crime must be checked, how great seems the guilt of those who intentionally use, to destroy life, the knowledge by which they are pledged to preserve it.

By our statutes, as at present standing, the mother can hardly be reached for her part in the crime. This should no longer be. Unless proved insane, the wretch who had caused the death of her offspring, perhaps by her own hand, should be made to suffer corresponding exposure and punishment. If married, as is too often the case, her crime should be considered as infinitely increased.

Much of the public indifference and error on the subject of criminal abortion is owing to the influence of certain misguided or brutal men, who by their publications or lectures, have given rise to a belief that the induction of abortion is alike the prerogative and duty of the married. Such offenders, whose guilt should be measured by their numberless victims, are at present beyond the reach of the law; but, as evident criminals, they should be punished. The community should be made to understand and to feel, that marriage, where the parties shrink from its highest responsibilities, is nothing less than legalized prostitution."

The Committee have so far achieved a triumph. At the last Boston meeting, their Report was warmly contested, but accepted by a close vote. At New Bedford, the debate being participated in by gentlemen from all parts of the State, many of whom are unmentioned in the brief report of the Boston Medical Journal it was evident that the majority of the Society was decidedly in their favor; and from the character of the men composing that committee, we are confident that they will not rest till they have secured all they desire. They aim at an open and general condemnation of the crime by the profession, and at thus removing the imputation now so generally considered as resting on its fair name. They aim at exposing, and even if better laws should be found valueless, at thus, to some extent at least, controlling and checking the crime.

In this connection, we cannot forbear noticing an infamous statement that appeared in a late number of the Boston Medical and Surgical Journal; it is from an anonymous stricture upon the Committees Report.

We are led to understand by the unsatisfactory excuse for the statement referred to, offered by the editors in a subsequent number of the Journal, that it was at once branded as a *libel upon the profession*, at the ensuing meeting of the Suffolk District Medical Society.

We quote the following:—

"Argue as forcibly as they may, the Committee will fail to convince the public that abortion in the early months is a crime, and a large proportion of the medical profession will tacitly support the popular view of the subject."

This assertion, taken in the sense most naturally suggesting itself to any one, would then seem no less than a deliberate falsehood, and would tend to prejudice the profession, wrongfully and dangerously, in public opinion; and when our brother editors admitted, in their apology, that they were for a moment, "willing to allow that this is capable of a construction which would imply a libel upon the profession," they assumed a position strangely at variance with that in their issue of December 13, 1855, when they so eloquently asserted that:—

"For ourselves, we have no fear that *the truth*, as told by the writer of this address (Dr. Storer's introductory) in reference to the crime of procuring abortion and the scarcely less heinous offence of preventing impregnation, would do aught but good in this, or in any city. It would appear that sheer ignorance in many honest people, is the spring of much of the horrible *intra-uterine murder* which exists among us; why not then enlighten this ignorance? It would be far more effectually done by some bold and manly appeal like that to which we allude, than by the private and scattered influence of honorable practitioners alone. In this case we will guarantee that vice will be all the more hated the more it was revealed, and would be neither pitied nor embraced.

The alarming extent of these evil practices is admitted; why attempt to conceal them any longer? Will not the mischief bye and bye be all the more deadly for delaying exposure and attempting relief?

If nearly every practitioner of medicine has his instances of application either to effect premature delivery or to prevent conception, what must the aggregate amount of these demands be? And how great reason have we for fully believing the ideas advanced by Dr. Storer, viz: that not only are felonious practices the source of the great diminution so visible in modern days in the families of the married; but also that the imperfect sexual connexion practiced both illicitly, and by husbands and wives, while it of course lessens our population, at the same time lays the foundation of many uterine diseases. This is at once an important and plausible suggestion. While perhaps, as yet, incapable of being substantiated by actual proof, the balance of evidence is decidedly in its favor, and on the most natural grounds. Whatever interferes with the full and proper exercise of any function, is likely to induce irregularity in its performance, and finally organic disease. This is at once philosophical and in accordance with the will of the Creator; *Leges naturæ non impune franges* If impregnation be prevented by the well known means so widely used, or in any way, why should we not look for the termination of the naturally aroused uterine excitement, which fails of its legitimate end, in congestion, inflammation, and final disorganization? This is a question of vital importance to any people—to all parents. In silence and by sufferance, these mighty evils are feeding on the life blood of the nation itself.

Whatever estimate may attach to our opinion, we believe that not only 'ought these not so to be,' but that the public should know it from good authority. Such an exercise of his knowledge, experience and true moral courage is not only the province but the conscientious duty of the Physician; expediency with its cold heart and leaden footed pace should be hooted from the path of usefulness and rectitude. To apprehend ill-effects and danger to public morals from telling the truth (especially when it has been too long waited for), is both a *petitis principii* and as a rule will not obtain. Rules, not exceptions, are our recognized guides."

The discussion has fairly begun, and the moral sense of the community cannot fail to be aroused and bettered. Already are the daily papers commenting on, and praising the manly course of the profession in Massachusetts, which we trust to see followed everywhere. The Boston Traveler thus closes a long article in its editorial columns, on the New Bedford debate.

" Public attention should be drawn to this last resort for getting rid of the cost and the unsatisfactoriness of young families. If all our people want to be rich, no poor folks; if all would have the luxury without the woe of life, let us take some other method than this spartan and barbaric retrogression. It would be almost as well to sink into the slough of Utah, and be 'sealed ' with the polluting caresses of the Deacons of Brigham Young's temple of productiveness, as for the mothers of our land habitually to stain their souls with the smothering of infant life. Its dangers in every and all stages are not properly understood. It is one which should be thought of. Among all the mischiefs which have arisen from our gregariousness in cities and in hotels, none is more terrible than this; terrible to the mother, terrible to society, terrible to all concerned."

We feel that we may well be pardoned for first speaking at length as we have, of the connection of our Massachusetts brethren with this subject, as the war seems first to have fairly begun with them; and will only say in addition, that we have personal knowledge that the evil is no less prevalent in our community. We have in this City, a woman engaged in this nefarious occupation and with whom our authorities are uuwilling to meddle, on account of her personal popularity as a *devoutly pious person*, and we have daily evidences that to pracure abortion is one of the first desires of a large proportion of pregnant women. We have no pardon to ask for the length of our article, for we feel it to be a subject too long neglected, and no longer to be winked out of sight. and we will be glad to publish any case which may be presented us, where Abortion has been produced, or attempted.

Dr. H. R. Storer has taken a stand in this matter alike creditable to his head and his heart, and we feel that he will receive the hearty thanks of every *true* physician.

That his efforts are appreciated by the profession at large, is evident, from the fact that he was appointed by the American Medical Association at its late meeting at Nashville, as Chairman of a Special Committee "on Criminal Abortion, with a view to its general suppression," to report next year at Washington, and may be regarded as an earnest, that the work now begun, *will be carried forward.—New-Hampshire Jour. of Medicine.*

